# Immunological Landscape and Molecular Therapeutic Targets of the Tumor Microenvironment in Hepatocellular Carcinoma

**DOI:** 10.3390/ijms26167836

**Published:** 2025-08-13

**Authors:** Yusra Zarlashat, Abdul Ghaffar, Flora Guerra, Anna Picca

**Affiliations:** 1 Department of Biochemistry, Government College University Faisalabad, Faisalabad 38000, Pakistan; yusrazarlashat@gcuf.edu.pk (Y.Z.); aghaffaruaf@yahoo.com (A.G.); 2 Department of Biological and Environmental Sciences and Technologies (DiSTeBA), University of Salento, 73100 Lecce, Italy; flora.guerra@unisalento.it; 3 Fondazione Policlinico Universitario “A. Gemelli” IRCCS, 00168 Rome, Italy; 4 Department of Medicine and Surgery, LUM University, 70010 Casamassima, Italy

**Keywords:** immune cells, immunosuppression, immune checkpoint inhibitors, immune evasion, immunotherapy, liver cancer

## Abstract

Hepatocellular carcinoma (HCC) is the most common liver cancer, with poor survival rates in advanced stages due to late diagnosis, tumor heterogeneity, and therapy resistance. The tumor microenvironment (TME) in HCC has a crucial role in tumor progression, characterized by a complex interaction of immune cells, stromal components, and immunosuppressive signaling pathways. Chronic inflammation driven by viral infections, metabolic dysfunction, and alcohol consumption triggers an immunosuppressive TME, promoting immune evasion and tumor growth. Immune cell populations, such as myeloid-derived suppressor cells, regulatory T cells, and tumor-associated macrophages, contribute to immunosuppression, while cytotoxic T lymphocytes and natural killer cells exert anti-tumor effects. Recent advances in immunotherapy, mainly immune checkpoint inhibitors (ICIs) targeting programmed death-ligand 1 and programmed cell death protein 1 and cytotoxic T-lymphocyte-associated protein 4, have revolutionized HCC treatment, though response rates remain limited. Combined therapies using tyrosine kinase inhibitors, anti-angiogenic agents, and ICIs improve patient outcomes. This review discusses the immunological mechanisms contributing to HCC progression, the role of immune cell subsets in tumor evasion, and therapeutic interventions, from conventional treatments to advanced immunotherapies. Ongoing clinical trials, barriers to effective treatment, and future directions to enhance HCC management and patient survival will also be overviewed.

## 1. Introduction

Hepatocellular carcinoma (HCC) is the common primary liver cancer, arising from hepatocytes, and represents 90% of liver cancer cases [1]. Despite improvements in treatment and diagnosis, HCC is still quite severe, with minimal survival rates, particularly in advanced stages, due to late diagnosis, tumor heterogeneity, and resistance to traditional treatments. The five-year survival rates for HCC vary significantly by stage: 25.9% to 41.7% for early-stage cases, dropping to 5.9% for intermediate-stage, and falling further to just 0.2–0.4% for advanced-stage [2]. Risk factors of HCC include chronic viral hepatitis, hepatitis B virus (HBV)/hepatitis C virus (HCV), metabolic dysfunction-associated steatotic liver disease (MASLD), and chronic exposure to alcohol, aflatoxin, and tobacco [3,4]. Besides these causal agents, several intrinsic factors, including genetic mutations, tumor microenvironment (TME), epigenetic modifications, and clonal evolution of cancer cells, also play a major role in the development of HCC [5]. Non-resolving liver inflammation is a major risk factor for the occurrence of liver cancer. About 90% of HCC patients experience chronic inflammation, leading to fibrosis, cirrhosis, and finally HCC [6]. The TME, a dynamic network of immune cells, stromal components, and aberrant signaling pathways that collectively enable immunosuppression, angiogenesis, and tumor metastasis, contributes to HCC [7]. The liver contains various immune cell populations, such as myeloid-derived suppressor cells (MDSCs), T cells, and tumor-associated macrophages (TAMs) that cluster together during tumor development inside the tumor immune microenvironment (TIME), fostering an immunosuppressive milieu. Conversely, natural killer (NK) cells, cytotoxic cluster of differentiation (CD) 8^+^ T cells, and proinflammatory CD4^+^ T cells with the T-helper type 1 (Th1) phenotype interact to prevent pro-tumor effects and provide effective anti-tumor responses [8]. Recent advances in immunotherapy, particularly immune checkpoint inhibitors (ICIs) targeting cytotoxic T-lymphocyte-associated protein 4 (CTLA-4), programmed death-ligand 1 (PD-L1), and programmed cell death protein 1 (PD-1), have revolutionized HCC treatment. However, response rates remain limited due to primary and acquired resistance. Combined therapies using tyrosine kinase inhibitors (TKIs), anti-angiogenic agents, and ICIs show potential in enhancing clinical outcomes [2]. This review provides a comprehensive overview of the immunological mechanisms involved in tumor evasion and discusses the latest advances in therapeutic interventions from conventional treatments to cutting-edge immunotherapies. A discussion of the main ongoing clinical trials, as well as challenges in treatment resistance, and future directions will be provided, highlighting the need for biomarker-driven personalized therapies to improve HCC patient survival.

## 2. Tumor Microenvironment in Hepatocellular Carcinoma

The TME consists of cancer cells as well as cancer-associated fibroblasts (CAFs), stromal cells, endothelial cells, and innate and adaptive immune cells. The TME is closely linked to tumor formation, survival, metastasis, and progression of HCC [9]. Research has also focused on developing treatments that target processes such as angiogenesis, infiltration, and tumor cell adhesion in TME. While earlier studies focused on the role of adaptive immune cells due to the strong cancer-fighting potential of T lymphocytes, current therapies targeting the TME mostly focus on the body’s innate immune responses. These treatments are chimeric antigen receptor (CAR) T cell therapies blocking immune checkpoints. Current immunotherapies use monoclonal antibodies targeting CTLA-4 and PD-1 checkpoints to block immune system inhibition [10].

The liver displays a unique form of immunotolerance, characterized by the continuous presentation of antigens to peripheral leukocytes in the absence of costimulatory signals from liver-resident cells, including hepatocytes, Kupffer cells (KCs), and dendritic cells (DCs). KCs secrete interleukin-10 (IL-10), which promotes the growth of regulatory T cells (Tregs). These interactions ensure that the liver maintains immune tolerance, preventing unnecessary immune responses that may lead to tissue damage. This balance between immune activation and suppression within the TME and the liver is pivotal for maintaining both effective tumor immunity and liver homeostasis [11]. However, in a precancerous microenvironment (PME), this becomes disrupted. The PME, which arises in inflammation, chronic liver injury, and fibrosis, triggers a pro-inflammatory environment that initiates carcinogenesis. In this altered microenvironment, the ratio between pro-inflammatory cytokines interleukin (IL)-2, IL-12, IL-15, and interferon-β (IFN-β) and anti-inflammatory cytokines, tumor growth factor (TGF)-β, IL-10, IL-13, is perturbed, thus contributing to immune evasion, tissue remodeling, and chronic inflammation. These factors collectively promote the transformation of the PME into a tumor supportive TME, further promoting carcinogenesis. Therefore, the PME acts as an early factor in cancer development, whereby the immune and stromal conditions support tumor formation and progression [12].

As the PME progresses, it gradually transitions into the TME, with immune cells like Tregs, MDSCs, and TAMs infiltrating the TME. These cells contribute to the development of an immunosuppressive environment that promotes cancer progression. In contrast, immune cells, including NK cells, cytotoxic CD8^+^ T cells, and pro-inflammatory Th1 CD4^+^ T cells, respond to tumor growth by blocking pro-tumor effects. However, immunosuppressive cells and cytokines in the TME further promote the progression of the tumor, highlighting the relevance of the immune landscape in the PME to TME transition [13].

## 3. Immune Cells in Hepatocellular Carcinoma

**Figure 1:**
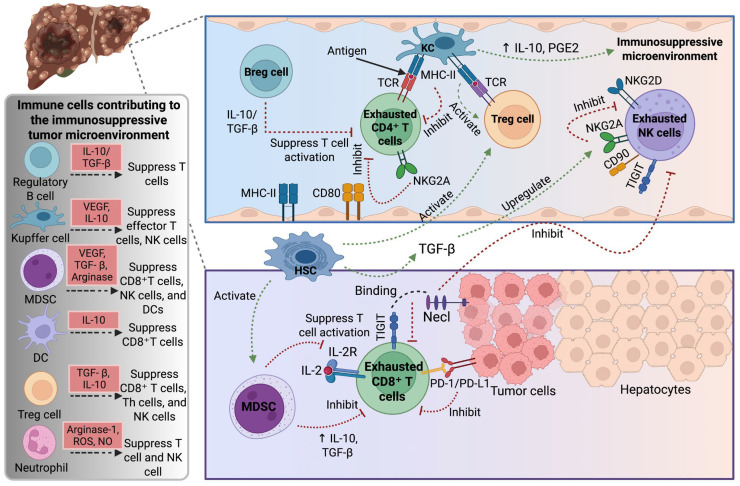
Schematic representation of the contribution of immune cells to the immunosuppressive tumor microenvironment. Key players such as Bregs, Tregs, MDSCs, KCs, and DCs secrete immunosuppressive cytokines like IL-10 and TGF-β, which suppress effector T and NK cell activity. Tumor-infiltrating CD8^+^ and CD4^+^ T cells, along with NK cells, become exhausted through the upregulation of inhibitory receptors (e.g., PD-1, TIGIT, NKG2A), limiting their anti-tumor functions. HCC cells and hepatic stellate cells further promote immune suppression via PD-L1 expression and TGF-β signaling. Together, these interactions create a permissive environment for tumor growth and immune evasion. Abbreviations: Breg, regulatory B cell; DC, dendritic cell; CD, cluster of differentiation; CD8^+^ T, cluster of differentiation 8^+^ T cells; CD4^+^ T, cluster of differentiation 4^+^ T cells; HSC, hepatic stellate cell; IL, interleukin; IL-2R, IL-2 receptor; KC, Kupffer cell; MDSCs, myeloid-derived suppressor cells; MHC-II, major histocompatibility complex class II; Necl, necrostatin-1; NK, natural killer; NKG2A/D, natural killer group 2 member A/D; NO, nitric oxide; ROS, reactive oxygen species; PD-1, programmed death protein 1; PD-L1, programmed cell death ligand 1; PGE2, prostaglandin E2; TCR, T cell receptor; TGF-β, transforming growth factor-β; TIGIT, T cell immunoreceptor with Ig and ITIM domain; Th, T-helper; Treg, regulatory T cell; VEGF, vascular endothelial growth factor. Created with BioRender.com.

Innate and adaptive immunity are crucial in immunosurveillance and cancer regulation. Immune activation and inactivation in the TME are regulated by the interaction of anti-tumor effectors and suppressors (Figure 1). The immune microenvironment is shaped by a dynamic balance between immunosuppressive cells (e.g., Tregs, MDSCs) and effector cells (e.g., cytotoxic CD8^+^ T cells, NK cells), which collectively establish an immunoregulatory niche that promotes local immune tolerance (tolerogenesis) [14]. MDSC and Treg cells are involved in immunosuppression and contribute to HCC immune evasion [15]. These cell types, along with dysfunctional CD8^+^ T cells, DCs [16], regulatory B cells (Breg cells), and neutrophils, among others [17], attenuate anti-HCC immune surveillance and effector functions.

### 3.1. T Cells

Cytotoxic CD8^+^ T cells are crucial in the anti-tumor immune response in HCC and prevent excessive inflammation in TME [18]. CD4^+^ T-helper cells also have an essential role in the immune response. CD4^+^ T-helper cells are activated by IL-12 and dendritic cell-derived type-1 interferon to produce proinflammatory (Th1) cytokines that stimulate anticancer immune response and cytotoxic T lymphocyte (CTL) proliferation. Upregulation of Th1 cytokines (IL-2, IL-1α, IL-1β, IFN-γ) promotes the initiation and effector functions of immune cells within the TME, leading to tumor control and a positive prognosis [19].

In HCC, T cell exhaustion develops gradually over time due to persistent antigen exposure in the TME. Tumor-specific CD8^+^ T lymphocytes gradually become activated and increase when exposed to tumor antigens [20]. However, prolonged stimulation overexpresses inhibitory receptors (PD-1), initiating the process of exhaustion. In this setting, CD8^+^ T cells develop a severe exhausted phenotype with inhibitory receptors, including T cell immunoreceptor with Ig and ITIM domain (TIGIT), lymphocyte-activation gene 3 (LAG-3), and CTLA-4. As a result, effector activities, including cytokine production and cytotoxicity, gradually decrease [21]. The presence of exhausted CD8^+^ T cells in the liver and the circulation is linked to a poor HCC prognosis [22]. Activation of the PD-1 receptor on T cells recruits Src homology region 2 domain-containing phosphatase-2 (SHP-2), which dephosphorylates signaling molecules of T cell receptor (TCR) signaling. This dephosphorylation disrupts downstream signaling cascades initiated by the phosphoinositide 3-kinase/protein kinase B signaling pathway, important for T-cell survival, proliferation, and effector functions [23].

Elevated PD-1 [24] and PD-L1 expression are associated with poor HCC prognosis [25]. T cell exhaustion is a reversible state, and exhausted T cells can be restored with checkpoint blockade under immunotherapy [26]. PD-1 blockade therapy improves outcomes in HCC patients [27]. In a phase III trial, 413 patients with advanced HCC who had not responded to sorafenib were assigned to receive a placebo (*n* = 135) or 200 mg of pembrolizumab (anti-PD-1) (*n* = 278) on day 1 of every 21-day cycle for a maximum of 35 cycles [28]. Pembrolizumab therapy decreased the risk of mortality and increased the median overall survival (OS) to 13.9 months from 10.6 months for the placebo. Patients responded to anti-PD-1 therapy if they had a greater baseline ratio of less-exhausted tissue-resident memory T cells (TRM) to severely exhausted T cells [21]. However, exhaustion is a complex state driven by multiple inhibitory pathways, so targeting PD-1 alone is often insufficient for complete reversal [26]. The CTLA-4 binds to CD80/CD86, and it antagonizes the interaction between CD28 and these ligands, inhibiting the co-stimulatory signal crucial to activate T cells. This results in decreased cytokine production, T cell proliferation, and survival [29]. In another study, Tremelimumab, an immunoglobulin G2 (IgG2) monoclonal antibody that targets CTLA-4, was administered to 21 patients with HCV-associated HCC at around 15 mg/kg every three months until the tumor progressed. With a median time to progression (TTP) of 6.5 months and a disease control rate of 76.4%, the responder rate was 17%. Effectively, by blocking immunological checkpoints, the therapy showed antiviral and anticancer effects. This also resulted in a positive treatment option in advanced HCC patients, with fewer adverse effects [30].

#### 3.1.1. Regulatory T Cells

Tregs are immunosuppressive CD4^+^ T cells defined by their CD4^+^CD25^+^ forkhead box protein P3 (FOXP3)^+^ phenotype. Elevated Treg levels in HCC correlate with tumor progression, reduced survival, and HCC severity [31]. The differentiation of Treg cells is triggered by TGF-β, IL-10, and cyclooxygenase-2 by tumor and stromal cells [32]. The combined effects of Tregs on DCs and cytotoxic T lymphocytes (CTLs) establish a strong immunosuppressive environment that supports tumor growth and progression. Tregs decrease CD80 and CD86 expression on DCs, preventing T cell activation. Simultaneously, by releasing suppressive cytokines and depleting IL-2, Tregs inhibit the expansion and function of CTLs [33]. Treg cell depletion is linked to enhanced anti-tumor immunity, inducing a reduction of CD8^+^ T cells by substantial chromatin remodeling. Gao et al. showed that C-C chemokine receptor type 4 positive Treg, overexpressing IL-10 and IL-35, are the predominant cells in HBV-related HCC, resulting in sorafenib resistance and high HBV viral load [34]. Treg cells are also known for their high stimulation of inhibitory signaling molecules and their direct cytolytic activity against antigen-presenting cells, directly via granzymes [35].

Certain subsets of type 1 regulatory cells and CD4^+^ T-helper cells produced when IL-10 is present from intra-tumoral myeloid cells use cytotoxic T lymphocyte-inhibitory functions and perform a similar role in the context of HCC [36]. Higher T-helper type 2 (Th2) cytokines (IL-4 and IL-10) in HCC are linked to HCC metastasis and progression, likely due to IL-4-initiated recruitment of TAM, leading to vascular endothelial growth factor (VEGF) and TGF-β secretion, contributing to tumor aggressiveness and metastasis [37].

The higher expression of Th2 cytokines in HCC is of quantitative significance, with IL-10 levels exceeding 12 pg/mL correlating with poor outcomes [38]. Additionally, IL-4 and IL-5 contribute to enhanced tumor aggressiveness by promoting TAM recruitment and subsequent cytokine secretion [39]. Th17 subsets, which produce IL-17, have also been detected in HCC, and their increased intra-tumoral presence is associated with reduced OS due to their role in promoting angiogenesis [40]. However, evidence regarding Th17 cells remains conflicting, with some studies indicating anti-tumoral activity while others reporting pro-tumoral effects [41]. These contradictory findings may indicate that Th17 cells contribute to HCC progression in a context-dependent manner, influenced by various factors including cytokine milieu (IL-17, IL-21, and IL-22), immune cell interactions (NK cells and CD8^+^ T cells), tumor cell signaling, microbiota, genetic, and epigenetic factors within the TME [42]. Th17 cells contribute to anti-tumor immunity in HCC by stimulating the activity of CTLs and NK cells by producing pro-inflammatory cytokines. This immune activation helps to control tumor growth, particularly in early-stage cancers [43]. Understanding the factors that influence Th17 cell behavior in the TME represents a key aspect for developing targeted therapies that control their effects, potentially improving outcomes for HCC patients.

#### 3.1.2. CD8^+^ T Cells

CD8^+^ T cells are CTLs, which are widely recognized as the predominant effectors in anti-tumor immunity. Circulating CD8^+^ T cells regulate the function of CD8^+^ tissue-resident memory T cells, important for effective HCC treatment [44]. However, the CD8^+^ T lymphocytes targeting neoantigens show resistance due to the presence of PD-L1^+^ LAMP3^+^ (lysosomal-associated membrane protein 3) DCs moving between the lymph nodes [45]. HCC is infiltrated by non-conventional T cells, which identify tumor antigens such as butyrophilin subfamily 3 member A1 (BTN3A1), a ligand activating γδ T cell receptors, without major histocompatibility complex (MHC) restriction. These properties have paved the way for new venues in immunotherapy [46]. The prognosis of HCC patients is influenced by the characteristics of CD8^+^ T cells. The increase of killer cell lectin-like receptor subfamily B (KLRB) (also called CD161) indicates that cytotoxic CD8^+^ T cells with an innate-like characteristic are linked to a poor prognosis [47]. In contrast, patients with viral-related HCC have an improved prognosis when their CD8^+^ T cells are activated and produce chemokine X-C motif chemokine ligand 1 (XCL1), which enhances the tumor-attacking immune response by activating other immune cells [48]. Clinically, PD-1 immune checkpoint suppression increases the function of tumor-specific CD8^+^ T-cells, reducing the tumor [49]. In another trial, 32 patients with advanced HCC received six doses of tremelimumab (anti-CTLA-4 antibody) at dosages of 3.5 and 10 mg/kg once every 30 days. Patients completed radiofrequency ablation (RFA) on day 36. An OS of 12.3 months indicated that tremelimumab has anticancer effects by increasing the CD8^+^ T cells in TME [50].

In HBV-related HCC, an immune imbalance between CD8^+^ and Treg cells promotes HCC tumor progression, causing a significant loss of anti-tumor effect of CD8^+^ T cells. ICIs are more effective when a proper balance between CD8^+^ effector T cells and immunosuppressive Treg cells in TME is maintained. Yan et al. showed that HBV-related HCC patients had varying levels of PD-1^+^ CD8^+^ cells and inducible T-cell COStimulator (ICOS)^+^ Tregs at different HCC stages. ICOS is a co-stimulatory molecule expressed on activated T cells, including Tregs, that modulates immune responses. Patients with a greater proportion of PD-1/ICOS^+^ Tregs showed a better clinical outcome, with less CD8^+^ T cell exhaustion. Additionally, their T cells are more active, with a better potential to proliferate and kill cancer cells [51]. Nine patients with early-stage HCC were given combination immunotherapy for up to two years in a neoadjuvant randomized study. Ipilimumab (anti-CTLA-4, 1 mg/kg on day 1 every 1.5 months) and nivolumab (anti-PD-1, 3 mg on day 1 of a 2-week cycle) were administered. Among them, three patients (33.3%) achieved a complete response. The treatment also enhanced T-cell infiltration in the TME, specifically increasing CD8^+^ T-cell populations, which contributed to an enhanced immune response [52].

### 3.2. Natural Killer Cells

Natural killer cells represent approximately 30% of hepatic lymphocytes, which is notably higher than their 5–15% presence in peripheral blood [53]. The number of CD16 and CD56 cell surface receptors makes NK cells homogenous. Liver resident NK cells (lrNK), which are supported by C-C chemokine receptor type 5 (CCR5) and C-X-C chemokine receptor type 6 (CXCR6), are CD16^dim^ and CD56^bright^ and are found in narrow-walled sinusoids alongside natural killer T cells (NKT), KCs, and T-cells. NKT cells are innate-like T lymphocytes that recognize lipid antigens and rapidly produce cytokines to modulate immune responses [54]. KCs and hepatocytes in the liver produce IL-2, IL-12, and IL-18, activating and increasing circulating NK cells and lrNK cells, which are CD16^dim^ CD56^bright^ in the enlarged liver [55]. Activated NK cells function quickly and independent of antigen presentation, release cytotoxic granules and generate cytokines (IFN-α and tumor necrosis factor-γ (TNF-γ)), to activate other immune cells. NK cells bring target cell death by expressing FAS receptor (CD95) and TNF-related apoptosis-inducing ligand (TRAIL) on target cells, triggering apoptotic signaling pathways within the target cell [56].

**Figure 2:**
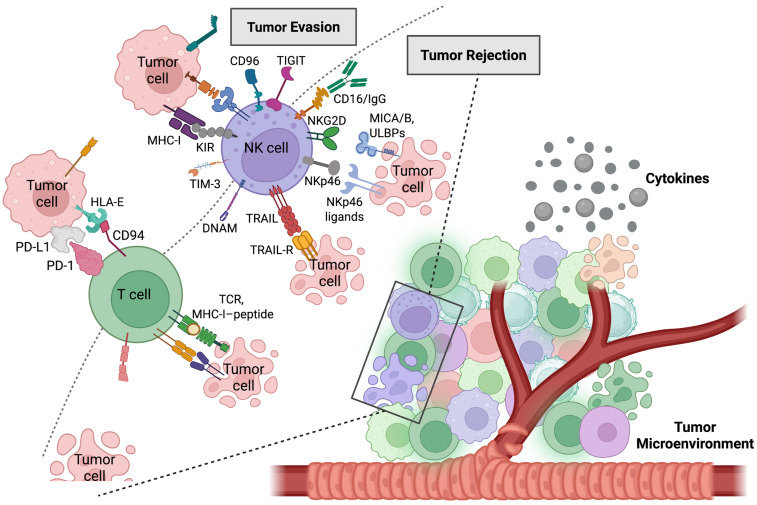
Illustration of the NK cell functional network in hepatocellular carcinoma. The interaction of inhibitory and activating signals is crucial in regulating NK cell recognition and eradication of tumor cells in HCC, serving as pivotal determinants in the immune response against HCC. Abbreviations: CD, cluster of differentiation; DNAM, DNAX accessory molecule; HCC, hepatocellular carcinoma; HLA-E, human leukocyte antigen E; IgG, immunoglobulin G; KIR, killer-cell immunoglobulin-like receptor; MHC, major histocompatibility complex; MICA/B, major histocompatibility complex class I-related Chain A and B; NK, natural killer; NKG2D, natural killer group 2, member D; NKp46, NK cell p46-related protein; PD-1, programmed death protein 1; PD-L1, programmed cell death ligand 1; TCR, T cell receptor; TIGIT, T cell immunoreceptor with immunoglobulin and ITIM domain; TIM-3, T-cell immunoglobulin and mucin-domain containing-3; TRAIL, tumor necrosis factor (TNF)-related apoptosis-inducing ligand; TRAIL-R, TRAIL-receptor; ULBP, UL16 binding protein. Created with BioRender.com.

The NK cell activation depends on stimulatory receptors that respond to various signals such as self-ligands, viral antigens, and tumor cells [57]. Natural killer group 2, member D (NKG2D) is an activating receptor expressed on immune cells (e.g., NK, NKT, γδ T, CD8^+^ T cells) that recognizes stress-induced ligands on infected or cancerous cells, initiating cytotoxic activity [58]. The NKG2D receptor is bound by MHC-I chain-related proteins A and B, as well as special long 16 (UL-16)-binding proteins [59]. Increased NKG2D receptor and its ligands expression is known in chronic hepatic disorders linked to metabolic diseases, hepatitis viral infections, and HCC [60]. Various receptors, such as NKG2D, natural cytotoxicity receptor (NCR), NK cell p44-related protein (NKp44), NKp30, NKp46, Fc gamma receptor III (FcγRIII), and DNAX accessory molecule-1 (DNAM-1), activate NK cells. DNAM-1 is triggered by Nectin/Nectin-like molecule (Necl) adhesion molecules, which activate and enhance the cytotoxic functions of NK cells. Necl interacts with CD96 on NK cells and TIGIT on both CD8^+^ T and NK cells to decrease DNAM-1 co-stimulation and NK function [61] (Figure 2). HCC expresses high levels of Necl and Nectin-4 levels, which are associated with poor prognosis [62]. TGF-β performs a role in immune regulation, particularly by inhibiting the activity of NK cells. One of its mechanisms involves the downregulation of NKG2D receptors, which are essential for cytotoxic function against tumor cells. TGF-β regulates the inhibitory receptors, including CD96 and TIGIT, on NK cells, while NKG2A/CD94 is expressed on both NK cells and T cells [63].

Studies have reported a depletion of peripheral blood NK cells in HCC patients, suggesting that the reduction, exhaustion, and dysfunction of NK cells are involved in HCC progression. This finding has prompted the exploration of NK cell-based immunotherapies against HCC in preclinical and clinical contexts. In a phase 1 clinical trial involving 11 HCC patients, researchers evaluated combined hepatic arterial infusion chemotherapy (HAIC) with locoregional high-dose autologous NK cell therapy. The treatment showed a 63% overall response rate, comprising complete responses in 36% of patients (4/11) and partial responses in 27% (3/11). The remaining 18% (2/11) showed either progressive or stable disease. Median OS reached 41.6 months, with progression-free survival (PFS) at 10.3 months [64].

### 3.3. Myeloid Cells

MDSCs and TAMs are the main myeloid cells in TME. TAMs are only tissue-resident cells, while MDSCs are immature myeloid cells found in cancer tissues and the circulation [65]. Endothelial cells and hepatic stellate cells (HSCs) produce chemokine (C-X-C motif) ligand 12 (CXCL12), which enhances intra-tumor myeloid cell migration by binding with the CXCR4 receptor, promoting tumor growth and immune suppression [66]. MDSCs reduce the level of arginine in the TME by producing arginase, thus inhibiting T cell proliferation. Additionally, they promote the synthesis of TGF-β and IL-10, which expands the Treg cells and stimulates PD-L1 expression on effector T cells, which enhances inhibitory signaling [67]. TAMs represent the majority of tumor-infiltrating leukocytes in HCC, and their occurrence is strongly linked with poor prognosis [68]. Further, depletion of CD8^+^ T cells and MDSC in HBV-related HCC cases after liver resection resulted in a decrease in tumor diameter [69]. Infiltrating monocytes and resident macrophages in the TME adopt an immune-regulatory phenotype in response to high IL-10 production by MDSCs. This phenotypic shift leads these cells to produce growth factors (GFs), i.e., TGF-β and VEGF, helping in tumor progression, which activate nuclear factor kappa-light-chain-enhancer of activated B cells (NF-κB) to promote cancer cell stemness, and the synthesis of matrix metalloproteinases to support metastasis formation [70]. A phase 1 trial recruited 36 HCC patients receiving sorafenib as a standard treatment and studied the CCAAT/enhancer-binding protein alpha (C/EBPa) in tumor development. Patients were treated with C/EBPa small activating RNA (MTL-CEBPA) that showed reduction of tumors and a decrease in peripheral blood monocytic-MDSCs [71]. Like MDSCs, TAMs also reduce the proliferation of anti-tumor cytotoxic T-cells while promoting regulatory CD4^+^ T-cell growth by expressing PD-L1 on their surface, secreting TGF-β and IL-10, and producing nitric oxide and arginase.

### 3.4. Cancer-Associated Fibroblasts

Cancer-associated fibroblasts are important in the HCC microenvironment, with activated fibroblasts being the most common form. They promote tumor development and metastasis by secreting inflammatory cytokines, GFs, and chemokines, while fostering an immunosuppressive microenvironment [72]. Recent research suggests that CAFs are a heterogeneous group of cells with diverse origins and functions in TME [73]. CAFs originate from cells such as HSCs, neutrophils, MDSCs, pericytes, adipocytes, endothelial cells, and tumor cells, going through epithelial-mesenchymal transition (EMT) [74]. CAFs have phenotypic and functional diversity, exhibiting both pro-tumorigenic and anti-tumorigenic properties [75]. A phase Ib/II study identified the critical role of fibroblast growth factor receptor 4 inhibitor (BLU-554) combined with anti-PD-L1 in locally advanced HCC patients. The findings, which showed a 100% disease control rate, supported the use of BLU-554 as a therapeutic adjuvant [76].

Activation of stem cell signaling pathways can contribute to CAF-mediated therapy in some cases. In a TME, a soft matrix rigidity promotes tumor growth by enabling cell spreading, while a stiffer extracellular matrix (ECM) increases cancer stem cell (CSC) multiplication and self-renewal as HCC progresses [77]. CAFs interact with cancer cells to reinforce their cancerous properties [78]. CAF precursors are attracted to tumor cells, and regular fibroblasts develop into CAFs. TMEs are produced when CAFs release growth factors and cytokines, which increase the proliferation of HCC cells and resistance to treatment. The TME is modified, and lymphocyte recruitment is controlled by the chemokine-chemokine receptor (CK-CKR) network [79]. Research evidence indicate that CAFs increase tumor-metastatic characteristics and raise the levels of C-C motif chemokine ligand 2 (CCL2), CCL5, CCL7, CCL26, and CXCL17.

## 4. Etiological Influences of Tumor Microenvironment

HCC displays considerable heterogeneity from both molecular and clinical standpoints, largely due to its diverse etiologies, including alcohol consumption, hepatitis infections, and non-alcoholic steatohepatitis (NASH), which drive ongoing hepatocyte injury and regeneration. This heterogeneity is present in both tumoral and non-tumoral components of the disease. Unraveling the complex relationships among etiology, disease progression, and molecular features remains a major challenge [80]. The progression of HCC is heterogeneous, whereby internal factors, such as chromosome instability and genetic mutations, are tightly linked to immunosuppressive TME. The TME is determined by the expression of viral antigens and etiopathogenesis, promoting tumor neoantigen evasion from immune surveillance [8]. Prolonged viral infections stimulate abnormal adaptive immunological responses and pro-inflammatory innate immune cells to induce hepatocarcinogenesis [81]. This process creates a pro-fibrotic and pro-inflammatory environment, which in turn promotes genetic mutations and alterations in cellular pathways, ultimately resulting in the malignant transformation and development of HCC [82]. Understanding the different etiological contributions to HCC is important to develop targeted prevention strategies, early diagnostic methods, and effective therapies against HCC.

### 4.1. Immune Landscape in Virus-Related HCC

HBV and HCV promote hepatocarcinogenesis through distinct mechanisms, including chronic liver inflammation with a compromised antiviral immune response, oxidative stress induced by viral and immunological proteins, and the disruption of cell signaling pathways by viral proteins [83]. When HBV infects a liver cell, its relaxed circular DNA (rcDNA) converts to closed circular DNA (ccDNA), serving as a template for viral replication. During this process, fragments of HBV DNA incorporate into the host genome, contributing to a common early stage of chronic HBV infection [84]. Long-term HBV infection causes HCC due to an ineffective CD8^+^ T-cell response that sustains necroinflammation, with HBV potentially triggering immune responses that are both pro- and anti-carcinogenic. CD8^+^ T cell infiltration shows a dual role, contributing to inflammation and liver damage while participating in immunological surveillance as well. This process could be counteracted by antiplatelet therapy, a novel approach that inhibits platelet activity to reduce the pro-carcinogenic effects of chronic inflammation, potentially lowering the risk of HCC [85]. A trial enrolling HCC patients who had liver resections (442 HCC patients with platelet therapy, and 1768 patients without platelet therapy) concluded that platelet therapy was associated with higher OS and recurrence-free survival [86].

**Figure 3:**
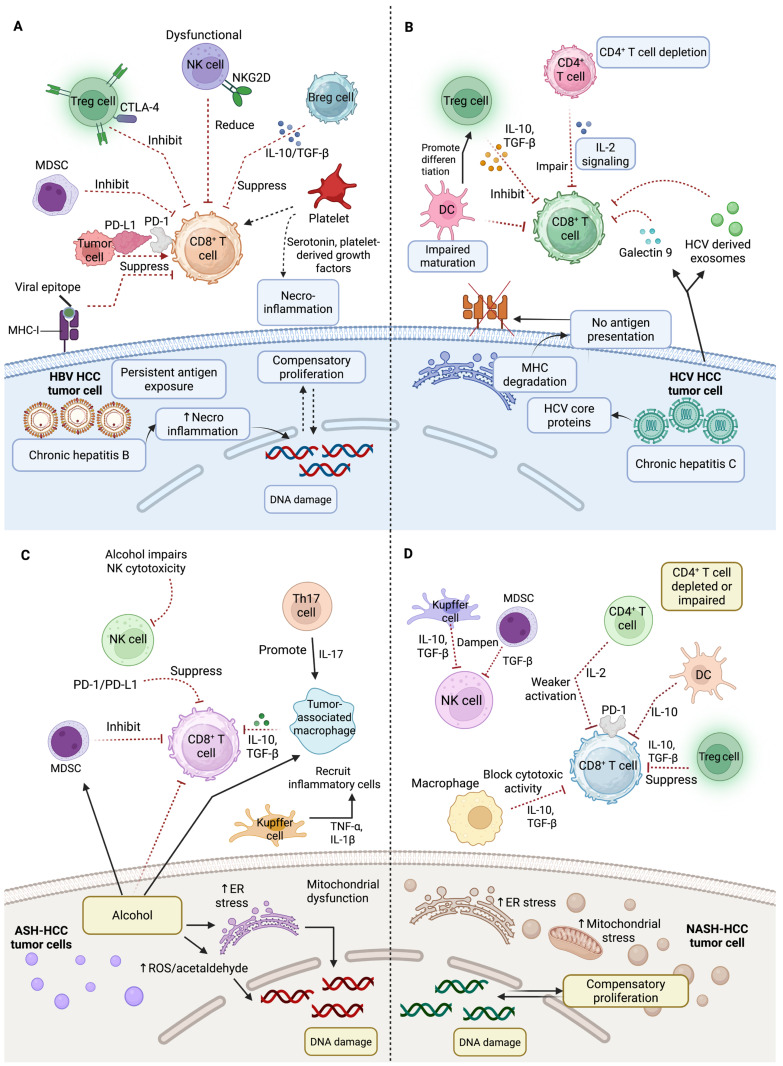
Immune response dynamics in HCC. (**A**) In HBV-related HCC, persistent viral epitopes, and immune checkpoint activation (PD-1/PD-L1, CTLA-4) trigger CD8^+^ T cell exhaustion, while dysfunctional NK cells, Bregs, and MDSC create an immunosuppressive milieu that promotes viral persistence and tumor growth. (**B**) In HCV-related HCC, chronic antigen exposure induces CD8^+^ T cell exhaustion and dysfunctional CD4^+^ T cell support. Meanwhile, Tregs and tolerogenic DCs suppress cytotoxic immunity through IL-10 and TGF-β, promoting fibrosis and HCC development. (**C**) In alcohol-related HCC, alcohol metabolism, and gut-derived endotoxins trigger KCs activation and inflammatory cytokine release, while TAM, Th17-driven IL-17 production, and immune checkpoint signaling suppress CD8^+^ T cell and NK cell function, promoting fibrosis and tumor progression. (**D**) In NASH-related HCC, lipotoxic hepatocyte injury and chronic inflammation activate KCs and Th17 cells while recruiting TAMs, MDSCc, and Tregs. These changes suppress CD8^+^ T and NK cells via PD-1/PD-L1 and IL-10/TGF-β signaling, impairing immune surveillance and driving fibrotic, pro-tumor conditions. Abbreviations: ASH, alcoholic steatohepatitis; Breg, regulatory B cell; CD8^+^ T cell, cluster of differentiation 8^+^ T cells; CD4^+^ T cell, cluster of differentiation 4^+^ T cells; CTLA-4, cytotoxic T-lymphocyte-associated protein 4; DC, dendritic cell; ER, endoplasmic reticulum; HBV, hepatitis B virus; HCC, hepatocellular carcinoma; HCV, hepatitis C virus; IL, interleukin; KC, Kupffer cell; MDSC, myeloid-derived suppressor cells; MHC-I, major histocompatibility complex class I; NASH, nonalcoholic steatohepatitis; NK, natural killer; NKG2D, natural killer group 2, member D; PD-1, programmed cell death protein 1; PD-L1, programmed cell death ligand 1; ROS, reactive oxygen species; TAM, tumor-associated macrophage; TGF-β, transforming growth factor-β; Th, T-helper cell; Treg, regulatory T cell. Created with BioRender.com.

HCC cells lack full HBV antigens but have short mRNAs that produce epitopes activating HBV-specific T cells. Clinical examples of this effect include tumor regression observed with the use of autologous T cells with genetic modifications that express T cell receptors designed to target these epitopes [87]. However, persistent HBV infection alters the hepatic immune infiltrate, producing a tolerogenic environment that limits antitumor immunity, due to dysfunctional HBV-specific T cells and multiple active inhibitory mechanisms within the chronically inflamed liver [88]. In chronic HBV infection, NK cells and T cells are dysfunctional due to multiple inhibitory mechanisms within the liver, including the PD-1 and T-cell immunoglobulin and mucin-domain containing-3 (TIM-3) [89], poor antigen-presenting cell activity, over-expression of co-inhibitory ligand (PD-L1), the release of immunoregulatory cytokines including TGF-β and IL-10, and an increased regulatory cell ratio [88]. Compared to non-viral HCCs, Treg cells and CD8^+^ resident memory T cells that are abundant in HBV-related HCC exhibit higher PD-1 expression and functional exhaustion, which results in a poorer prognosis. Immunosuppression by PD-1 Treg is reversed with anti-PD-1 therapy, where Treg correlates with poor prognosis, and CD8^+^ memory T cells stay in liver and effectively attack tumor cells, therefore having a better prognosis [90] (Figure 3A).

HCV primarily infects hepatocytes through entry receptors, although it also affects other cells and organs [83]. Clathrin-mediated endocytosis allows the virus to reproduce and translate its RNA in the endoplasmic reticulum, producing a polyprotein that breaks into structural and non-structural proteins [83]. Mutations in core protein isoforms enhance HCC risk through epigenetic changes, chronic inflammation, liver fibrosis, and cirrhosis, direct oncogenic effects of viral proteins, and immune evasion [83]. Direct-acting antivirals eliminate HCV, which is not incorporated into the host genome, lowering HCC incidence. Still, a higher risk of HCC continues to exist even after the successful elimination of HCV infection [91]. In a phase III study, 743 advanced HCC patients were treated with nivolumab against sorafenib [92]. The OS did not approach statistical significance, although the median OS for nivolumab was 16.4 months, whereas that of sorafenib was 14.7 months. Notably, nivolumab demonstrated a higher response rate and a better safety profile, with fewer side effects. Subgroup analyses indicated clinical benefit in patients with hepatitis B or C, and sorafenib-naive and intolerant individuals. These findings suggest that nivolumab offer a clinically meaningful alternative to sorafenib in selected patient populations [92]. An increasing demand exists for additional preventive strategies to reduce the ongoing inflammatory, epigenetic, metabolic, and pro-oncogenic processes [91]. HCV evades the immune system by producing dysfunctional and exhausted CD8^+^ T cells, targeting tumor cells to become exhausted. These exhausted CD8^+^ T cells are defined by increased PD-1 and TIM-3, decreased immune molecule interferon-gamma (IFNγ) production, and cluster of differentiation 127 (CD127) expression. This immune evasion helps HCV to persist in the patient’s body [93]. HCV-infected cells generate exosomes that induce monocytes to secrete galectin 9, which promotes CD8^+^ T cell TIM-3 expression, prolonging this dysfunctional state [17]. Moreover, HCV infection is associated with a decrease in CD4^+^ T cells producing IL-2 and an increase in immunosuppressive CD4^+^CD25^+^ Tregs [94]. Additionally, the virus-specific CD8^+^ T cells release IL-10 to decrease immune response throughout chronic HCV infection, and the development of hepatocarcinogenesis [95]. Viral mutations helps the virus to evade immune system detection [93] as mutated HCV core proteins stop the presentation of viral antigens to immune cells via MHC I molecules [96]. These processes contribute to immune evasion associated with HCV-related HCC (Figure 3B).

Advancements in immunotherapy strategies have emerged from insights into the metabolic constraints that viruses impose on HCC. HBV-related T lymphocytes are inhibited in HBV-related liver damage by granulocytic MDSCs depleting arginine, an essential nutrient for T cell function [97]. Dysregulated cholesterol metabolism is also a hallmark of HCC. Genes involved in cholesterol biosynthesis, uptake, efflux, and esterification (e.g., *3-hydroxy-3-methylglutaryl-CoA reductase*, *squalene epoxidase*, *sterol regulatory element binding protein 2*, and *sterol O-acyltransferase 1*) are often upregulated in HCC, leading to increased intracellular cholesterol that supports rapid tumor cell growth and division [98]. Some experimental models show that cholesterol overload increases the activation of pro-oncogenic signaling pathways (e.g., signal transducer and activator of transcription 3, c-Jun N-terminal kinase, and protein kinase B) and alters membrane dynamics, both of which favor migration and invasiveness of HCC cells [99]. High levels of the esterification enzyme sterol O-acyltransferase 1 (SOAT1) in certain HBV-related HCCs disrupts cholesterol balance, which, in turn, promotes tumor cell migration and proliferation. SOAT1 inhibitors reduce these effects on HBV/HCC-specific tumor-infiltrating cells, potentially reducing tumor progression [100].

### 4.2. Immunological Landscape of Non-Viral HCC

Chronic liver inflammation and hepatic steatosis may occur upon heavy alcohol use, a high-calorie diet, and a sedentary lifestyle [101]. NASH-induced inflammation is linked to inflammatory cells being spread throughout the liver tissue, while viral hepatitis has structured inflammatory foci and localized areas where immune cells are clustered around infected liver cells to form distinct lesions [102].

Alcohol intake is responsible for 30% of the worldwide burden of HCC. Alcohol consumption regulates the permeability of the gut, leading to translocation of pathogen-associated molecular patterns (PAMPs), including microbial products (lipopolysaccharides). These PAMPs produced by immunomodulatory microbiota pass through the intestinal barrier and enter the liver. In the liver, PAMPs activate stellate cells and inhibit immune responses, highlighting the function of gut microbiota and intestinal permeability in developing liver diseases such as fibrosis, cirrhosis, and ultimately HCC [103]. Alcoholic steatohepatitis (ASH) inhibits the liver T cell recruitment and accumulates pro-tumorigenic, immunosuppressive granulocytic MDSCs to support liver damage and increase HCC risk [104]. In contrast, aflatoxin B1 (AFB1) exposure promotes a distinct immunosuppressive TME by inducing aryl hydrocarbon receptor activation and subsequent PD-L1 expression in hepatocytes. This highlights a different mechanism of immune evasion in AFB1-associated HCC compared to other etiologies, such as NASH or ASH [105]. The infiltration of neutrophils into the liver parenchyma, increased by heavy alcohol consumption, disrupts immune balance, and leads to the release of neutrophil extracellular traps (NETs). However, secondary antigen stimulation reduces NET formation, weakening antibacterial defenses. At the same time, impaired macrophage clearance of NETs prolongs liver inflammation and injury, ultimately affecting the hepatic immunological milieu and contributing to increased inflammation and liver damage [106]. ASH is likely correlated with alterations in the immune cells, along with changes in the hepatic cytokine milieu, which hinder the immune response to HCC [104]. Activated macrophages and KCs in ASH produce pro-inflammatory cytokines (IL-1β, TNF-α, IL-6), causing fibrosis and damage to the liver [107]. While decreased NK cell activity limits the potential of neutrophils to target and eliminate malignant cells, including HCC, they also increase tissue damage via reactive oxygen species and enzymes [108] (Figure 3C).

Recent studies have shed light on the molecular mechanisms driving the progression of NASH to HCC, highlighting the intricate interplay between the fibrogenic, inflammatory, and metabolic pathways. These findings indicate that NASH-induced HCC involves an immune cell–hepatocyte network associated with metabolic stress, fibrogenesis, and chronic inflammation [109] (Figure 3D). Platelets have a crucial role in initiating inflammatory processes associated with steatosis, thus contributing to the progression of NASH and HCC from NASH [110]. Platelets accomplish this through the platelet-specific glycoprotein Ib-α (GPIb-α) and the interaction between KCs, platelets, and inflammatory monocytes to initiate the inflammatory processes associated with steatosis, NASH, and HCC [110]. Antiplatelet therapy in 722 HCC patients (control n: 111, treatment n: 611) showed significant improvements in OS, with a negative correlation with liver-associated deaths. Further, in HCC patients receiving transarterial chemoembolization (TACE) as first-line therapy, antiplatelet therapy showed prevention of tumor progression [111]. Certainly, both therapeutic and prophylactic interventions with antiplatelet therapy exhibit a reduction in NASH and NASH-induced HCC [110].

The development of NASH-induced liver cancer is influenced by other immunological responses, including resident KCs’ activation as well as leukocyte recruitment to the liver, including NK, neutrophils, monocytes, and natural killer T cells. These cells contribute to inflammation through the release of chemokines, cytokines, eicosanoids, nitric oxide, and reactive oxygen species; therefore, these cells get metabolically activated in both NASH and HCC through intricate interactions with hepatocytes [112]. The suppression of hepatocyte metabolic machinery such as lipid oxidation, due to direct and indirect interactions with immune cells, leads to increased metabolic stress, endoplasmic reticulum stress, and mitochondrial stress. These stress responses contribute to NASH progression and its potential for HCC [113]. It has been shown that the liver affected by NASH gradually accumulates worn-out, abnormally activated CD8^+^ PD-1^+^ T cells. Targeting PD-1 with therapeutic immunotherapy in preclinical models of NASH-induced HCC increased the number of activated CD8^+^ PD-1^+^ T cells inside tumors; however, it did not cause tumor regression, indicating that tumor immune surveillance was compromised [16]. Notably, non-viral HCCs, especially those related to NASH, exhibit a lower response to immunotherapy compared to viral HCCs. Auto-aggressive CD8^+^ PD-1^+^ T cells establish a pro-tumorigenic environment within the TME by inhibiting tumor surveillance function [114]. γδ T cells produce IL-17A, which performs a crucial role in both NASH and HCC progression by promoting inflammation through chemokine production in hepatocytes, attracting neutrophils, and depositing fatty acids in the liver. Targeting the NKG2D pathway in γδ T cells presents a good therapeutic method for managing NASH and HCC [115] (Figure 3D).

## 5. Pathways and Signaling Cascades Mediating Immune Evasion

Signaling pathways related to the tumor also have an effect on the immune microenvironment in HCC. Mutations in the catenin b1 (*CTNNB1*) gene and the activation of wingless/integrated (Wnt)/β-catenin signaling pathway decrease CCL5 expression and prevent the recruitment of DCs [116]. Consequently, these mechanisms facilitate immune evasion and reduce the response to ICIs. The β-catenin pathway also suppresses the NKG2D ligand expression on HCC cells, therefore weakening NK cell-mediated MHC-independent immune responses [117]. TGF-β signaling shows a role in the progression of HCC and contributes to pro-tumorigenic and immunosuppressive cancer effects. The high expression of cellular myelocytomatosis oncogene (*MYC*), observed in approximately 50–70% of HCC patients, also triggers PD-L1 expression [118]. Mutations in the tumor protein p53 (*TP53*) gene are also a common finding in HCC, with approximately 40% of these cancers showing such alterations [119].

### 5.1. Role of Wnt/β-Catenin Signaling in HCC Tumorigenesis and Immune Evasion

The Wnt/β-catenin signaling pathway preserves liver homeostasis and is involved in HCC development. β-catenin is phosphorylated and targeted for destruction when Wnt ligands are not detected. Wnt ligands attach to frizzled receptors to stop β-catenin from degrading, allowing its accumulation in the cytoplasm and entrance into the nucleus [120]. β-catenin interacts with lymphoid enhancer-binding protein transcription factors (TFs) and T-cell factor for activating genes that support cell proliferation and survival, including matrix metalloproteinases (*MMPs*) and *MYC* [121]. This pathway also promotes *MMP* expression and causes EMT, which makes tumor invasion more accessible. Wnt/β-catenin signaling is abnormally activated in HCC, which leads to the proliferation of cancer stem cells (CSCs). Tumor growth and survival are promoted by continuous activation of β-catenin, which is produced by *CTNNB1* gene mutations. β-catenin signaling promotes the invasive and migratory abilities of HCC cells by triggering EMT, a process that enables cancer cells to gain stem-like traits and resist apoptosis. Wnt/β-catenin signaling also supports macrophage polarization towards the M2 phenotype in the TME, which is linked to immunosuppression and tumor development. M1 macrophages normally have anti-tumor activities, but M2 macrophages promote tumor growth and metastasis [120].

### 5.2. TGF-β Signaling in HCC Progression and Immune Suppression

The non-canonical and canonical signal transduction molecules of the TGF-β superfamily (SMAD)-dependent pathway involves Rho-like GTPases and phosphoinositide 3-kinase/protein kinase B signaling are the two main pathways through which TGF-β signals. TGF-β frequently promotes tumor growth in HCC patients, especially if the disease is more advanced. TGF-β is known to increase the invasive characteristics of HCC cells, promoting tumor metastasis.

The expression of the TF FOXP3 by Tregs, which have developed from naïve CD4^+^ T cells, based on TGF-β is a common finding. The signaling pathways of SMAD2 and SMAD3 mediate this process. Although Tregs are important for immunological homeostasis, tumors frequently use them to inhibit anti-tumor immunity. High TGF-β levels in the context of HCC support Treg growth in the TME, which enhances immune suppression and promotes tumor progression. TGF-β signaling contributes to the expansion and activation of MDSCs, which have strong immunosuppressive effects. MDSCs secrete immunosuppressive molecules such as arginase 1, which depletes L-arginine, an essential amino acid to make T cells active. As such, MDSCs limit T cell activation and initiate tumor growth [122]. TGF-β affects macrophage polarization in the TME. It promotes monocyte differentiation into M2 macrophages, which are linked to immunological suppression and pro-tumorigenic activity. M2 macrophages release mediators that enhance tumor cell invasion and survival while inhibiting T cell responses [123]. The activation and proliferation of CTLs are negatively affected by TGF-β which induces a shift in the immune system to resist pro-inflammatory responses by suppressing the important TF expression required for Th1 differentiation. This enables cancers to evade immune surveillance [122]. A Phase III trial is evaluating livmoniplimab (an anti-leucine-rich repeat containing 32 (LRRC32)/TGF-β1 monoclonal antibody) plus a PD-1 inhibitor in advanced HCC. Given TGF-β’s dual role in tumors, biomarkers like SMAD7 and CXCR4, previously linked to pro-tumor effects of TGF-β, may help identify patients who benefit from TGF-β inhibition. Testing these gene clusters may improve targeted therapy selection [124].

### 5.3. Immune Checkpoint Expression and Immune Evasion in HCC

Hepatocytes, KCs, DCs, and tumor cells express PD-L1 and are crucial cells in HCC. By upregulating PD-L1, which binds to PD-1 on T cells and prevents its antitumor activity, tumor cells evade T cell attack and develop immunological tolerance. This results in decreased antitumor immunity and T cell exhaustion [125]. Studies have shown that drugs targeting the PD-1/PD-L1 pathway exhibit significant anti-HCC activity; however, only a few patients achieved complete remission with PD-1/PD-L1 inhibition. Enhancing anti-tumor immunity and improving outcomes can be achieved by combining PD-1/PD-L1 inhibitors with other therapies, i.e., CTLA-4 and anti-VEGF antibodies [126]. This observational study evaluated the use of atezolizumab (anti-PD-L1) plus bevacizumab (anti-VEGF) in 61 unresectable HCC patients who had already been treated with systemic therapy. Results showed a median OS of 16.2 months, with manageable adverse effects [127].

Hepatotropic viral infection in HCC and liver dysfunction have prompted the necessity for further investigation to establish the effectiveness of blocking PD-1/PD-L1 in HCC patients. In the phase I/II trial, nivolumab (0.1–10 mg/kg) was administered biweekly to advanced HCC patients with or without HBV/HCV using a 3+3 dose-escalation design. A 3 mg/kg dose was selected for 214 patients across viral and non-viral subgroups. Objective response rates were 15% in the escalation phase and 20% in the 214 patients, with an OS of around 15 months [128]. In a phase III study, patients with advanced HCC were randomized to receive sorafenib (TKI) 400 mg twice daily or cabozantinib (TKI) 40 mg once daily with atezolizumab (ICI, anti-PD-L1 antibody) 1200 mg every 21-day cycle. When compared to sorafenib alone, the cabozantinib + atezolizumab combination treatment considerably increased the median PFS (6.8 months vs. 42 months) [129]. Tremelimumab (anti-CTLA-4) was given to 32 advanced-stage HCC patients in a different experiment at doses of 3.5 and 10 mg/kg once every 30 days for six treatments; participants completed RFA on day three. An OS of 12.3 months indicates that tremelimumab enhances T-cell activation and increases the presence of intratumoral CD8^+^ T cells in TME, resulting in anticancer effects [50].

## 6. Conventional and Advanced Therapies for Hepatocellular Carcinoma Management

### 6.1. Curative and Localized Treatment Approaches

Curative strategies for HCC (liver transplantation, surgical resection, and local ablative therapies, i.e., microwave or radiofrequency ablation) vary according to tumor stage and liver. However, the applicability of liver transplantation is limited, with only about 5% of patients eligible. Surgical resection is also less favorable in older patients due to comorbidities; one study reported significantly lower OS in patients aged ≥65 years following surgical intervention [130]. Additional treatments, such as TACE, are commonly used in early/intermediate-stage HCC [131]. Stereotactic body radiation therapy has appeared as the best treatment, demonstrating better control and OS than TACE for medium-sized HCC tumors and serving as an effective alternative to RFA for unresectable cases with a low risk of local recurrence. TACE is a common treatment for intermediate-stage HCC. Older people (≥80 years) undergoing TACE demonstrated similar safety and effectiveness compared to younger patients, although they received fewer sessions on average (median of two sessions for older vs. six for younger patients) [132]. Immunotherapies and targeted therapies have expanded the treatment options for advanced, unresectable HCC [133]. The loco-regional and systemic therapies hold the potential for increasing patient survival in the context of liver dysfunction and HCC [134]. Thirty intermediate-stage HCC patients received treatment with lenvatinib 8 mg daily, whereas 60 patients with the same stage of HCC received TACE. In comparison to the TACE group, the lenvatinib group demonstrated superior therapeutic potential for intermediate-stage HCC patients, as evidenced by a longer median PFS (16.0 vs. 3.0 months), and a significantly improved OS (37.9 vs. 21.3 months) [135].

### 6.2. Systemic and Immunotherapeutic Approaches

Tyrosine kinase inhibitors play an important role in the systemic treatment of advanced HCC. TKIs are multi-targeted agents that inhibit tyrosine kinase receptors such as the vascular endothelial growth factor receptor (VEGFR) critical for tumor angiogenesis, proliferation (signaling pathways), and stromal support. This multi-kinase inhibition disrupts tumor vascularization and growth signaling pathways, which are dysregulated in HCC. For instance, sorafenib in advanced HCC targets kinases such as rapidly accelerated fibrosarcoma (RAF), VEGFR, and platelet-derived growth factor receptor (PDGFR) to inhibit tumor growth and angiogenesis, and induces an improvement in OS from 7.9 to 10.7 months compared to placebo [136]. Lenvatinib is another first-line TKI that inhibits VEGFR, fibroblast growth factor receptor (FGFR), PDGFR, and other kinases, showing OS non-inferior to sorafenib (13.6 vs. 12.3 months) with better PFS. Combination regimens of TKIs with ICIs or locoregional therapies (e.g., hepatic arterial infusion chemotherapy or TACE) are under investigation and show promising efficacy and safety in recurrent unresectable HCC [137].

Immunotherapy with ICIs (PD-L1/PD-1 and CTLA-4 inhibitors) enhances cytotoxic immune responses within tumors. These ICIs have transformed the therapeutic landscape of HCC, emerging as a promising and impactful treatment strategy. Results of a phase I/II study in patients with advanced HCC are available. Study participants were divided into three groups: a control group (people without viral infections), and HBV and HCV infection group. Each group received doses of nivolumab (anti-PD-1 antibody, 0.1–10 mg/kg) every 14 days. The objective response rates for patients receiving 3 mg/kg of nivolumab daily were 15% and showed that the tumor size shrank in all three groups [128].

Combination therapies improve the efficacy of conventional HCC treatments. The combination treatment of ICI with anti-VEGF/TKI demonstrated better results than monotherapy in advanced-stage patients. Sorafenib is approved for advanced HCC, with newer regimens such as atezolizumab (anti-PD-L1) plus bevacizumab (anti-VEGF), and tremelimumab (anti-CTLA4) plus durvalumab (anti-PD-L1) now established as standard treatments, and emerging therapies like tislelizumab (anti-PD-1) and pembrolizumab (anti-PD-1) combined with lenvatinib (TKI) under investigation [138]. In a two-year phase Ib study, 104 patients with incurable HCC received 200 mg of pembrolizumab every three weeks and 8 mg of lenvatinib (TKI) every two weeks. The tumor-inhibitory effects and higher survival rate of lenvatinib and pembrolizumab combined are shown by a decrease in tumor size, an OS of 22 months, and a median PFS of 9.5 months [139]. In a phase I/II trial, cabozantinib (TKI) 40 mg daily with nivolumab (anti-PD-1) 240 mg on the first day after 2 weeks (double immunotherapy) or nivolumab 3 mg/kg after 2 weeks plus cabozantinib 40 mg daily, with ipilimumab (anti-CTLA-4) 1 mg/kg after 1.5 months (triple immunotherapy) were given to HCC patients who had not responded to sorafenib. The doublet regimen demonstrated promising antitumor activity and a favorable safety profile, with a median OS of 20.1 months, while the triplet regimen achieved a median OS of 22.1 months [140].

Another phase 1b-2 trial evaluated tiragolumab (600 mg) plus atezolizumab (1200 mg) and bevacizumab (15 mg/kg) in 41 untreated unresectable HCC patients, compared to 18 patients receiving bevacizumab 15 mg/kg plus atezolizumab 1200 mg. The triplet therapy demonstrated higher response rates (43% vs. 11%) versus standard atezolizumab/bevacizumab, with manageable toxicity. These findings suggest TIGIT inhibition enhances PD-L1/VEGF blockade in HCC [141]. On the first day of 21-day cycles, patients with advanced-stage HCC were split into two groups in a phase 1b trial. One group received atezolizumab (anti-PD-L1) at a dose of 1200 mg along with bevacizumab (anti-VEGF) at a dose of 15 mg/kg. The other group received atezolizumab alone. The first group’s median PFS in this study was 5.6 months, whereas the second group’s was 3.4 months, suggesting that the combination treatment was more effective [142]. The combined use of TKIs-ICIs (cabozantinib + nivolumab +/− ipilimumab, pembrolizumab + lenvatinib, camrelizumab + rivoceranib, atezolizumab + cabozantinib, apatinib + camrelizumab), or ICIs-ICIs (durvalumab + tremelimumab, ipilimumab + nivolumab), and ICIs-VEGF inhibitors (sintilimab + bevacizumab, atezolizumab + bevacizumab) showed potential to treat advanced via preventing angiogenesis and affecting the infiltration of immune cells, suggesting the potential for better treatment results [2]. In Table 1 are reported ongoing and completed clinical trials investigating various systemic, cellular, and immunotherapeutic approaches for HCC, such as TKIs, ICIs, CAR-T therapies, and combination regimens.

### 6.3. Barriers and Advancements for Treatment

Treatment approaches are complicated by the significant tumor heterogeneity that characterizes HCC. Therapy response varies throughout tumor subtypes, making it challenging to predict therapy outcomes and efficiently adapt treatments. Genetic variants, environmental factors, and underlying liver diseases like cirrhosis are all susceptible to this heterogeneity. As a result, a general strategy is frequently ineffective, requiring specific therapies that adjust to the distinctive characteristics of every tumor [143]. Many patients are detected with HCC at advanced stages because of the asymptomatic nature of HCC in early stages and the limitations of current surveillance methods. A late-stage diagnosis greatly decreases the chances of a successful intervention [144]. Treatment resistance represents another challenge in HCC management. The expression of multidrug resistance (MDR) proteins on tumor cells can lead to the active efflux of chemotherapeutic agents, diminishing their efficacy. Furthermore, the TME increases resistance through several mechanisms, including hypoxia and the existence of cancer stem cells, which are more resistant to conventional therapies [145].

Treatment for HCC seems to have a promising future, with numerous clinical trials in progress aimed at overcoming current limitations. Investigating novel agents targeting specific HCC progression pathways, such as FGFRs and VEGF pathways. Trials are investigating the efficacy of various ICIs, both as monotherapies and combination therapy, to highly express the immune response against tumors [146]. Current research evaluates the potential benefits of integrating targeted therapies with immunotherapies and conventional treatments to enhance response rates and OS results. Clinical trials are increasingly incorporating biomarker analyses to identify suitable patients and customize treatments based on predicted responses. This could result in more effective and personalized care [147].

Recent research has revealed an unexpectedly high degree of heterogeneity in the immune cell populations within the TME of HCC. This complexity highlights the need for more personalized therapeutic approaches, as different immune landscapes may influence patient responses to treatment. Novel findings have shown the significant role of non-tumor cells, i.e., CAFs and endothelial cells, in modulating the immune environment of HCC. These cells have been found to contribute to immune evasion and tumor progression, providing new potential therapeutic targets. Emerging evidence suggests that the viral etiology of HCC, particularly hepatitis B and C infections, leads to distinct alterations in the TME. These alterations influence immune cell infiltration and response to ICIs, highlighting the need for etiology-specific treatment approaches. The application of spatial transcriptomics has discovered previously unknown spatial relationships between immune cells and tumor cells in TME. This technology has provided novel information on how the spatial organization of these cells influences tumor growth and response to therapy. Published studies have started to explore the combined effects of plant-derived compounds, such as curcumin and resveratrol, with ICIs in modulating the TME of HCC. These findings open new venues for combinatory treatments that could enhance the value of ICIs and overcome resistance in HCC patients.

## 7. Conclusions and Future Directions

The TME is crucial for HCC therapy, as it influences treatment response, progression, and resistance by modulating immune activity, angiogenesis, and stromal interactions. This highlights the importance of targeting TME components to enhance therapeutic effectiveness and improve patient outcomes. Combination of immunotherapy with anti-VEGF agents or TKIs in HCC has shown improved treatment efficacy and prolonged survival by simultaneously targeting multiple pathways. However, challenges remain, including treatment-related toxicity, the development of resistance, and the limited response rates observed in a significant subset of patients. Further research is needed to optimize therapeutic strategies, particularly by targeting immunosuppressive mechanisms within the TME, such as Tregs, MDSCs, and alternative immune checkpoint pathways, to overcome resistance and improve treatment outcomes.

Early-stage diagnosis of HCC is necessary for effective treatment and improved patient survival. Advanced immune profiling techniques such as CIBERSORT and single-cell RNA sequencing have revealed diverse immune cell populations within the TME of HCC, providing valuable information on tumor progression and therapeutic response. Furthermore, advancements in technologies such as spatial transcriptomics, machine learning, and artificial intelligence hold significant promise for enhancing early-stage diagnostic accuracy and personalizing treatment strategies in HCC, ultimately leading to better clinical outcomes. Studies are investigating how natural compounds may enhance the ICI efficacy by altering the immune landscape, reducing immunosuppressive elements within the TME, and initiating anti-tumor immune responses. Future research should explore novel molecules that could synergize with ICIs, investigate their mechanisms of action and validate their efficacy in clinical trials to advance therapeutic strategies for HCC management.

## Figures and Tables

**Table 1:** Selected clinical trials for immunotherapies in HCC.

Trial ID	Treatment	Trial Phase	*N* of Patients	Targets	Status
NCT05337137	Nivolumab and Relatlimab + Bevacizumab	Phase I/II	162	PD-1, LAG-3, VEGF	Active
NCT03412773	Tislelizumab vs. Sorafenib	Multiregional phase III	674	PD-1	Completed
NCT04826406	Camrelizumab plus Apatinib	Phase II	40	PD-1 receptor	Recruiting
NCT06560827	CT011 CAR-GPC3 T cells injection	Phase I	30	CAR T cells	Recruiting
NCT06144385	CAR-GPC3 T cells	Phase I	20	CAR T cells	Recruiting
NCT06084884	AZD5851	Phase I/II	84	CAR-T therapy directed against GPC3	Recruiting
NCT05652920	Ori-C101 Hepatic arterial infusion	Phase I/II	105	GPC3-directed chimeric antigen receptor-modified T cells	Recruiting
NCT05323201	fhB7H3.CAR-Ts + Fludarabine + Cyclophosphamide	Phase I/II	15	Human B7H3 CAR-T cells	Recruiting
NCT05155189	C-CAR031 + Lenvatinib + PD-1(L1) monoclonal antibody	Phase I	44	GPC3 armored CART cell injection (C-CAR031)	Recruiting
NCT06001567	Avatrombopag	Phase II	30	Platelets	Recruiting

Abbreviations: C-CAR031, autologous chimeric antigen receptor T Cells (CAR-T) therapy targeting glypican-3 (GPC3); CT011 CAR-GPC3, CARsgen Therapeutics*’* 011, autologous CAR-T-cell therapy targeting the GPC3; fhB7H3.CAR-Ts, fully human B7H3-specific chimeric antigen receptor T-cells; LAG-3, lymphocyte-activation gene 3; *N*, number; Ori-C101, autologous anti-GPC3-CAR T lymphocytes Ori-C101; PD-1, programmed cell death protein 1; PD-L1, programmed cell death ligand 1; VEGF, vascular endothelial growth factor.
